# Grand challenges: Actualizing the potential of the gut microbiome to address global nutrition challenges

**DOI:** 10.3389/frmbi.2023.1146827

**Published:** 2023-02-23

**Authors:** Tiffany L. Weir

**Affiliations:** Intestinal Health Laboratory, Department of Food Science and Human Nutrition, Colorado State University, Fort Collins, CO, United States

**Keywords:** probiotic, precision health, personalized nutrition, gut microbiota, nutrition

It has been less than 200 years since Pasteur, Lister, Koch, and others changed the theory and practice of medicine through experiments demonstrating the role of microbes in disease; and we once again find ourselves poised on the edge of a microbial revolution. In the early 2000’s, the development of high throughput sequencing technologies gave scientist the tools to characterize previously uncultured microbial life in environments such as the human gut. This resulted in a deluge of studies describing microbial communities in different environments and under varying conditions. It quickly became apparent that diet/nutrition is a critical regulator in the assembly, colonization, and maintenance of the gut microbiota (Voreades et al., 2014; Sheflin et al., 2017; Gentile and Weir, 2018). Furthermore, it has become clear that these microbial assemblages influence the maintenance of health and development of disease in humans. Early studies demonstrated that obesity, arguably one of the most prevalent chronic diseases of our time, was not only associated with specific microbial signatures but that it was caused, at least in part, by microbial influences on metabolism (Bäckhed et al., 2005; Ley et al., 2005; Turnbaugh et al., 2006). It is now believed that numerous non-communicable diseases (NCD), and even mental health, are causally linked to the microbes in our gut. In fact, 80% of global premature deaths from NCDs are due to cardiovascular disease, cancer, chronic respiratory diseases, and diabetes (World Health Organization, 2022), which have all been associated with disruptions to the gut microbiota.

So where do we go from here? The next challenge is to translate this growing body of scientific work into real-world solutions for public health. Despite the potential role of the microbiome in nutrition, weight regulation, exercise performance, mental health, and disease prevention/treatment, there are very few evidence-based, microbiota-targeted strategies for health optimization. One could argue that the ~60 billion USD global probiotic and prebiotic market is a concrete manifestation of advances in microbiome science. There are hundreds of human clinical trials demonstrating the safety- and sometimes efficacy- of probiotic bacteria. There are even novel strains and bioengineered probiotic organisms available to consumers that provide a range of activities from production of neurotransmitters (Dinan et al., 2013) to metabolizing alcohol (Lu et al., 2020). However, in many countries there are strict regulations limiting specific health claims and limited guidance on the types and doses of probiotic products to consume for intended benefits (Cunningham et al., 2021). In addition, probiotics have not been widely adopted by the mainstream medical community for clinical use, except in limited situations (Su et al., 2020). Furthermore, after over two decades of research, less than a handful of microbiota-based biotherapeutics are approved or nearing approval by the US Food and Drug Administration. In November 2022, Rebiotix’s standardized capsules for fecal microbiota transplants (FMT) were the first to gain approval from the FDA. Several other therapeutics are in the pipeline, with Seres Therapeutics’ drug for recurrent *Clostridium difficile* likely to be the next to gain approval (Mullard, 2022). The process that inspired these drugs, fecal microbiota transplant (FMT), is still considered an “investigational” approach for treating recurrent *C. difficile* infections in much of the world, despite evidence of efficacy. The investigational designation restricts patient access to the procedure (Scheeler, 2019), with a recent study in Europe suggesting that it is only conducted in about 10% of all patients in which this treatment is indicated (Baunwall et al., 2021). Thus, translational application of microbiome science remains a significant challenge.

A recent perspective published in Nature Medicine identified areas of microbiome science that have the greatest potential to impact public health (Wilkinson et al., 2021). Nutrition and diet were high on this list because they are factors that affect everyone. In fact, personalized/precision nutrition was identified as an area that would require the least amount of research and infrastructure to implement in human populations. Already, there are several commercial personalized nutrition platforms that are directly derived from microbiome research. In 2015, researchers at the Weizmann Institute published an exciting study that challenged the assumption that glycemic load was an intrinsic property of foods by demonstrating that post-prandial glycemic responses to the same foods differed among individuals (Zeevi et al., 2015). Importantly, by applying predictive algorithms to their clinical datasets, they were able to use fecal microbiota profiles to extrapolate individual glucose responses to specific foods. Similarly, the PREDICT studies, the largest personalized nutrition clinical trials to date, have also integrated diet and microbiome data to develop predicted responses to individual foods (Berry et al., 2020). The resulting commercial personalized nutrition platforms, Day2 and ZOE, provide individuals with information to help maintain glucose control. However, the massive databases and artificial intelligence generated by these platforms and others could eventually be applied more broadly to include applications such as microbiota-associated biomarker identification for diseases, personalized probiotic recommendations, and identifying potential responses to drug/diet interventions. Finally, the US National Institutes of Health has recently invested in a large research infrastructure that would leverage All of Us- an historic effort to gather data from a diverse population of ~1,000,000 people, to better understand individual responses to nutrition and diet. The Nutrition for Precision Health initiative will integrate clinical, metabolomic, genomic and microbiome data from both clinically fed and free-living individuals and is specifically designed to capture data from a diverse population. However, while personalized/precision nutrition has the potential to find unique solutions to health issues and address health disparities, its current implementation through various commercial platforms makes it inaccessible to many. Only through continued multi-disciplinary collaboration and widespread buy-in by medical professionals, insurance companies, and employers will the full potential of personalized nutrition be realized (Berciano et al., 2022), and this will require a concerted effort to elevate the quality, consistency, and transparency of human-related microbiome research.

Another promising application of microbiome research is the use of microbiota-targeted intervention foods to address malnutrition (Mostafa et al., 2020; Chen et al., 2021), pioneered by the lab of Dr. Jeffrey Gordon at the University of Washington in Missouri. His group has used clinical and pre-clinical translational models to identify microbial profiles that are associated with biological age in healthy, normally developing young children. They noted that children experiencing severe acute malnutrition displayed delayed progression and establishment of a stable gut microbiota relative to normally developing children in the same community, and that this microbial “immaturity” persisted after treatment with commonly used nutritional intervention foods (Subramanian et al., 2014). Furthermore, incomplete recovery of normal growth parameters was associated with persistent immaturity of the microbiota, even after intervention, which led them to hypothesize that the malnutrition was causally associated with these deviations from normal microbiota development (Blanton et al., 2016). Through careful experimentation they have been able to identify a core ecogroup of microbes that are associated with nutritional sufficiency during the first 2 years of life (Raman et al., 2019), as well as culturally appropriate intervention foods that stimulate the growth of these microbes (Gehrig et al., 2019). The results, several microbiota-targeted intervention foods, were recently compared with the standard ready-to-use supplementary food (RUTF) to treat toddlers in Mirpur, Bangladesh with moderate acute malnutrition. One of these intervention formulations compared favorably with the standard RUTF in terms of growth, but only the microbiota-targeted intervention altered plasma proteins associated with inflammation and neurodevelopment as well as repairing the gut microbiota (Chen et al., 2021). Although long tern follow up will be needed to establish whether these changes persist and are associated with improved outcomes in metabolism, immunity, and neurodevelopment in addition to improving linear growth, this approach could have profound impacts on a major global health challenge.

Advances in microbiota-directed interventions for personalized nutrition and malnutrition represent significant progress in the field, will undoubtedly improve countless lives, and provide a roadmap for future discovery and implementation. However, the efficacy of these interventions relies on the plasticity of the microbiome; which can also present a unique challenge. For example, personalized nutrition approaches use microbial signatures to identify individualized glycemic responses to different foods. However, if the microbiome structure is causally related to the glycemic responses rather than serving as a mere biomarker of these responses, then recommended dietary changes may alter the microbiota in such a way that the response to different foods changes. Therefore, it is important that continued mechanistic research is conducted to determine whether the microbiota is serving as a driving factor of metabolic responses. In addition, there is a need to identify the frequency with which retesting of the microbiota and re-evaluation of its interactions with nutrition must be conducted. There is also a disproportionate focus on food/supplement/diet interventions that can change the microbiome, but a more challenging and equally important aspect of research would be to identify interventions that convey both stability and resilience to the microbiome. Are there foods or supplements that improve microbial stability and make a microbiome less susceptible to disruption by external stressors? This is an important but largely unexplored question in nutrition-related microbiome research.

Finally, actualization of applications that harness the full potential of microbiome science as it relates to nutrition and health will require continued research and adoption of common standards for study conduct and reporting. Currently, many of the microbiome studies in humans suffer from small sample sizes and a lack of diversity among participants. While larger and more controlled studies are being conducted, these smaller studies are not without merit, particularly when similar studies can be collectively assessed to draw conclusions. Until recently a lack of reporting standards for microbiome-related human studies have hampered the reproducibility of research and limited our ability to analyze results across studies. This was recently addressed by the development of a tool called Strengthening The Organization and Reporting of Microbiome Studies or STORMS checklist (Mirzayi et al., 2021). STORMS is an easy-to-understand, 17-item checklist of minimum reporting requirement for human-associated microbiota studies, structured to follow the outline of most standard scientific manuscripts. Designed to improve reproducibility and consistency in reporting, STORMS can also be a helpful tool in evaluating manuscripts for publication. Therefore, the next challenge is to encourage the scientific community, including publishers, to adopt these standards. Widespread adoption of STORMS could increase real-world impact of microbiome researchers and aid in actualizing the potential of our research to improve human nutrition and health.

## Author contributions

TW researched and wrote this article. The author confirms being the sole contributor of this work and has approved it for publication.
